# Examination of the Potential for Adaptive Chirality of the Nitrogen Chiral Center in Aza-Aspartame

**DOI:** 10.3390/molecules181214739

**Published:** 2013-11-28

**Authors:** Samir H. Bouayad-Gervais, William D. Lubell

**Affiliations:** Département de chimie, Université de Montréal, 2900 Boul. Edouard-Monpetit, Montréal, QC H3T 1J4, Canada; E-Mail: samir.bouayad-gervais@umontreal.ca

**Keywords:** azapeptide, peptide mimic, aspartame, dynamic chirality, hydrazine, taste, crystal structure, nitrogen chirality, semicarbazide

## Abstract

The potential for dynamic chirality of an azapeptide nitrogen was examined by substitution of nitrogen for the α-carbon of the aspartate residue in the sweetener *S*,*S*-aspartame. Considering that *S*,*S*- and *R*,*S*-aspartame possess sweet and bitter tastes, respectively, a bitter-sweet taste of aza-aspartame **9** could be indicative of a low isomerization barrier for nitrogen chirality inter-conversion. Aza-aspartame **9** was synthesized by a combination of hydrazine and peptide chemistry. Crystallization of **9** indicated a *R*,*S*-configuration in the solid state; however, the aza-residue chiral center was considerably flattened relative to its natural amino acid counterpart. On tasting, the authors considered aza-aspartame **9** to be slightly bitter or tasteless. The lack of bitter sweet taste of aza-aspartame **9** may be due to flattening from sp^2^ hybridization in the urea as well as a high barrier for sp^3^ nitrogen inter-conversion, both of which may interfere with recognition by taste receptors.

## 1. Introduction

**Figure 1:**
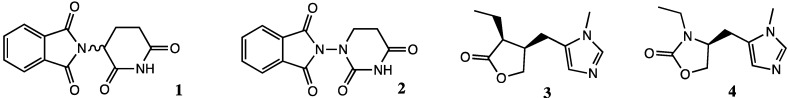
Thalidomide (**1**), pilocarpine (**3**) and their aza-analogs **2** and **4**.

Chiral nitrogen has typically a low isomerization barrier [1]. Substitution of a chiral carbon by nitrogen can thus create a dynamic chiral center in a molecule possessing properties of both enantiomers of its carbon counterpart. For example, replacement of the chiral carbon center by nitrogen in the drug thalidomide (**1**, Figure 1) gave aza-thalidomide **2** possessing activity comparable to its racemic mixture in which the inhibitory effects of the *R*-enantiomer on cyclooxygenase were tempered by the inactive *S*-isomer [2]. Alternatively, potential to adopt receptor requirements for recognition can give an aza-analog with potency similar to that of the more potent enantiomer. Such was the case when the chiral carbon at position-3 of pilocarpine (**3**) was replaced by nitrogen [3]. The resulting aza-pilocarpine **4** exhibited equal potency as the natural *S*-diastereoisomer, which is 10 times more potent than its *R*-isomer on muscarinic cholinergic receptors [4]. Moreover, the conversion of lactone **3** into cyclic carbamate **4** improved the duration of action of pilocarpine, likely due to suppression of inactivation by hydrolysis and ring opening. 

**Figure 2:**
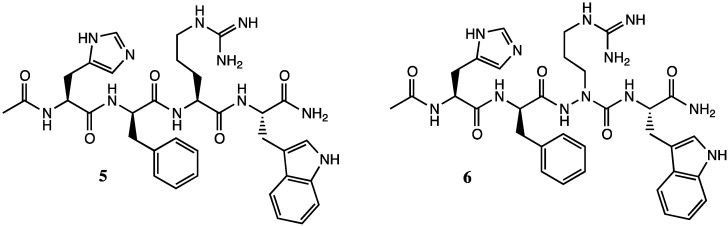
MSH tetra-peptide **5** and [aza-Arg^3^]MSH analog **6**.

Azapeptides in which nitrogen replaces the α-carbon of one of the amino acids in the sequence offer potential to have adaptive chirality, albeit the switch from amide to urea may flatten the chiral center if the sp^3^ nitrogen adopts sp^2^ character in resonance with the neighboring carbonyl [5]. Azapeptide analogs can thus show varying degrees of activity relative to their diastereomeric peptide counterparts. For example, in functional assays on HEK-293 cells stably expressing different mouse melanocortin receptor (MCR) subtypes (mMC1R, mMC3R, mMC4R, and mMC5R) replacement of aza-arginine for arginine in the melanocyte-stimulating hormone (MSH) tetra-peptide **5** gave [aza-Arg^3^]MSH **6**, which exhibited a drop of activity at the mMC1R that was less significant than that of the [D-Arg^3^]MSH isomer; however, more significant losses of activity than both isomers on the other receptor subtypes (Figure 2) [6].

**Figure 3:**
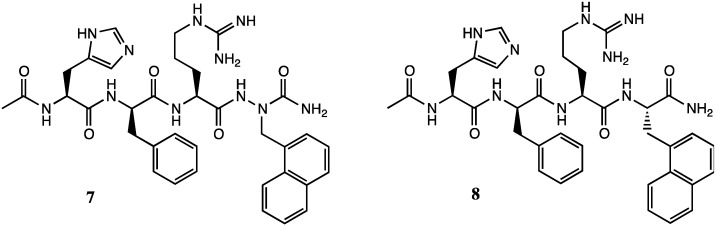
[aza-Nal-2^4^]MSH **7** and [L-Nal-2^4^]MSH **8** analogs.

On the other hand, replacement of 2-naphthylalanine by aza-2-naphthylalanine at position-4 of tetrapeptide **5** gave [aza-Nal-2^4^]MSH **7**, which exhibited respectively similar and significantly better potency as [L-Nal-2^4^]MSH **8** and its D-Nal-2^4^ diastereomer at all receptor subtypes (Figure 3) [6].

**Figure 4:**
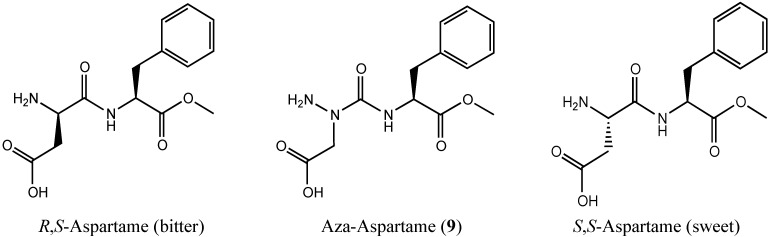
Aspartame isomers and [aza-Asp^1^]-aspartame **9**.

In the interest of further exploring the adaptive chirality of nitrogen in an aza-peptide analog, we selected to make an aza-analog of the sugar substitute aspartame, because earlier studies had demonstrated that the *S*,*S*- and *R*,*S*-diastereomers (Figure 4) exhibited sweet and bitter tastes, respectively [7]. Aza-aspartame **9** became an interesting target by which to judge the existence of adaptive chirality in an azapeptide, because of the potential for a bitter sweet taste by adaptation to the chirality of each diastereomer of the peptide counterpart at the different receptors on the tongue. The synthesis and author’s tasting of aza-aspartame **9** is now presented; moreover, crystals of **9** were obtained and examined by X-ray analysis, which has provided additional structural information on the character of the nitrogen center in aza-amino acid residues.

## 2. Results and Discussion

**Scheme 1:**
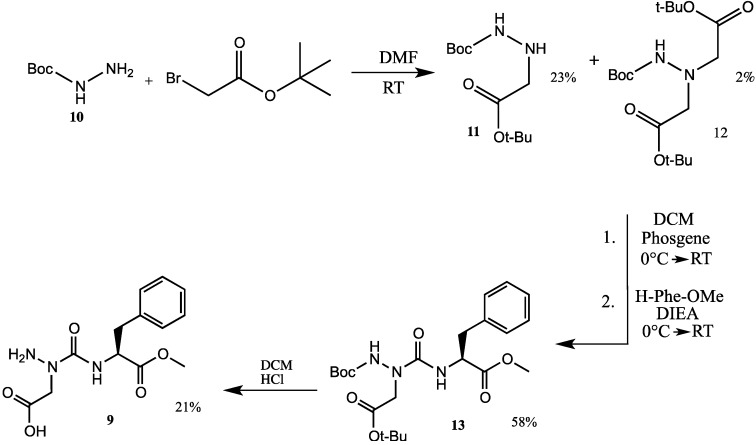
Synthesis of aza-aspartame **9** from *tert-*butyl carbazate **10**.

Aza-aspartame **9** was synthesized starting from *tert*-butylcarbazate (**10**) and *tert*-butyl bromoacetate (Scheme 1). Hydrazine ester **11** was obtained in 23% yield from the alkylation of carbazate **10** with the bromide (100 mol%) in DMF, after column chromatography. In addition, dialkylated product **12** (2% yield) was isolated and characterized by mass spectrometry. Activation of hydrazine **11** in toluene by dropwise addition of a solution of phosgene in toluene at 0 °C, followed by reaction of the resulting protected aza-aspartic acid chloride with phenylalanine methyl ester gave aza-dipeptide **13** in 58% yield after purification by flash chromatography [8]. Removal of the Boc group and *tert*-butyl ester was simultaneously achieved using HCl gas bubbles in dichloromethane. Aza-aspartame **9** was purified by preparative HPLC and crystalized from a methanol/water solution.

**Figure 5:**
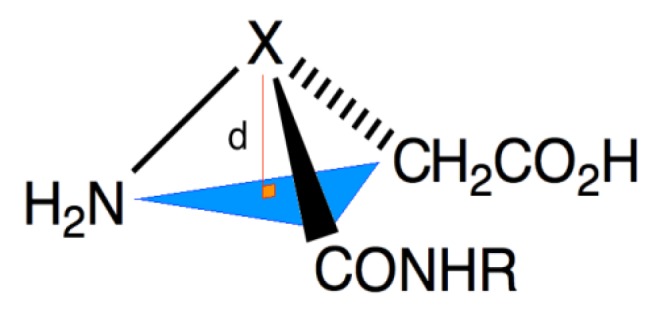
Measuring distortion from planarity in X-ray structure.

**Figure 6:**
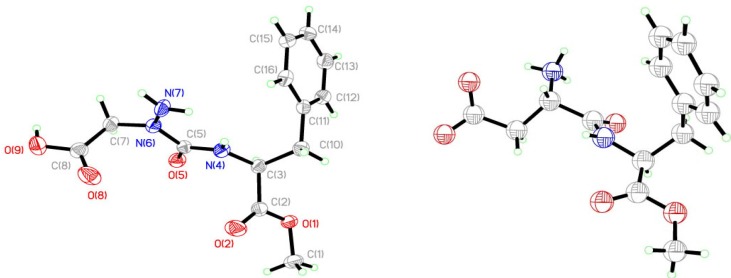
Aza-aspartame **9** and *S,S-*aspartame X-ray structures.

Analysis of the crystals of **9** by X-ray diffractometry provide a structure indicating the aza-amino acid nitrogen N(6) was nearly planar (Figure 5 and Figure 6). Examination of the distance between N(6) and the plane formed by N(7), C(5) and C(7) (Table 1) indicated a *R*-configuration with a slight 0.054(2) Å distortion from planarity, which was ten times smaller than that observed in the X-ray structure of aspartame [9]. The X-ray crystallographic data was deposited in the Cabridg (CCDC 972051) [10].

X is CH for *S*,*S*-aspartame, d = −0.54 Å (*S*-configuration) and X is N for Aza-aspartame, d = 0.054 Å.

Aza-aspartame (left) is almost planar at N(6) in contrast to *S*,*S*-aspartame (right) which possesses a tetrahedral geometry.

**Table 1:** Bond length and angles of **9**.

Bonds	Lengths [Å]		Angle [°]
C(1)-O(1)	1.4441(14)	C(2)-O(1)-C(1)	115.63(9)
O(1)-C(2)	1.3275(14)	O(2)-C(2)-O(1)	124.51(11)
C(2)-O(2)	1.2030(14)	O(2)-C(2)-C(3)	125.36(11)
C(2)-C(3)	1.5291(16)	O(1)-C(2)-C(3)	110.13(9)
C(3)-N(3)	1.4407(15)	N(4)-C(3)-C(2)	111.04(9)
C(3)-C(10)	1.5465(16)	N(4)-C(3)-C(10)	109.80(10)
N(4)-C(5)	1.3426(16)	C(2)-C(3)-C(10)	110.45(9)
C(5)-O(5)	1.2514(15)	C(5)-N(4)-C(3)	123.47(10)
C(5)-N(6)	1.3513(15)	O(5)-C(5)-N(4)	123.55(11)
N(6)-N(7)	1.4089(15)	O(5)-C(5)-N(6)	120.53(11)
N(6)-C(7)	1.4434(15)	N(4)-C(5)-N(6)	115.88(10)
C(7)-C(8)	1.5225(16)	C(5)-N(6)-N(7)	117.97(10)
C(8)-O(8)	1.2029(16)	C(5)-N(6)-C(7)	121.27(10)
C(8)-O(9)	1.3237(15)	N(7)-N(6)-C(7)	120.31(10)
C(10)-C(11)	1.5084(16)	N(6)-C(7)-C(8)	111.34(10)
C(11)-C(16)	1.3951(17)	O(8)-C(8)-O(9)	121.34(12)
C(11)-C(12)	1.3976(17)	O(8)-C(8)-C(7)	123.01(11)
C(12)-C(13)	1.3885(18)	O(9)-C(8)-C(7)	115.64(10)
C(13)-C(14)	1.3832(19)	C(11)-C(10)-C(3)	112.66(9)
C(14)-C(15)	1.3895(19)	C(16)-C(11)-C(12)	118.28(11)
C(15)-C(16)	1.3892(18)	C(16)-C(11)-C(10)	122.30(10)
		C(12)-C(11)-C(10)	119.42(10)
		C(13)-C(12)-C(11)	120.94(12)
		C(14)-C(13)-C(12)	120.15(12)
		C(13)-C(14)-C(15)	119.66(12)
		C(16)-C(15)-C(14)	120.18(12)
		C(15)-C(16)-C(11)	120.77(11)

**Figure 7:**
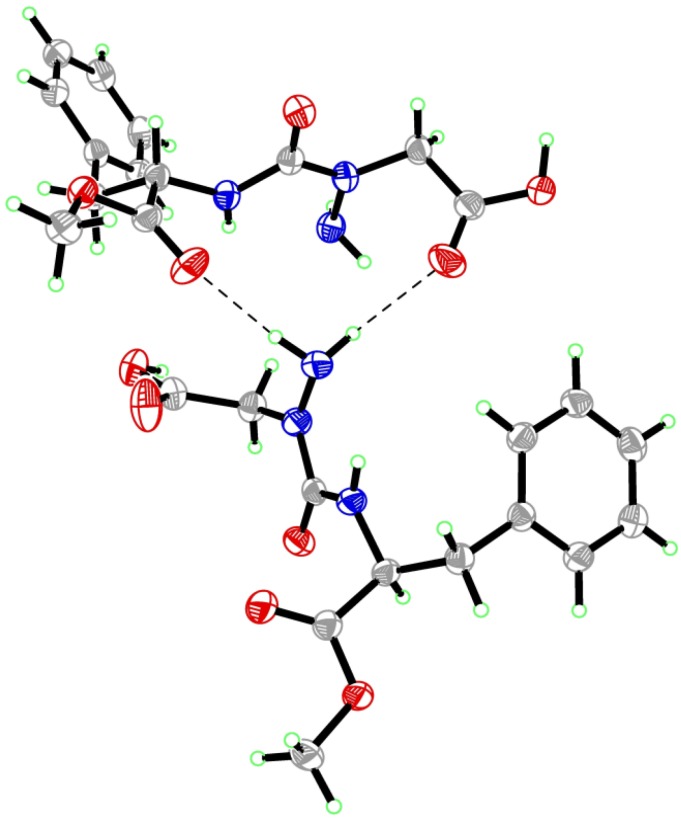
Packing of aza-aspartame **9** in the solid-state.

Examination of the crystal packing of **9** (Figure 7) shows that the amine terminal of the aza-Asp forms hydrogen-bonds with both the carbonyl oxygens of the aza-Asp β-carboxylate and Phe methyl ester of a neighboring aza-aspartame molecule. In this orientation, the lone-pairs of the neighboring hydrazine nitrogen sit perpendicular: that of the terminal amine points towards the hydrogen of the amine of its Phe residue and that of the chiral nitrogen projects away from the carbonyl of its aza-Asp β-carboxylate. Such packing may in part result from or explain why the semicarbazide nitrogen is distorted from planarity and adopts the *R-*(D)-stereochemistry. Isomerization and flattening of the nitrogen chiral center, due to changes in the state of conjugation of the nitrogen lone-pair with its neighboring carbonyl group, may occur in the absence of such crystal packing forces in solution. Although slow inter-conversion of the nitrogen stereocenter may give rise to diastereotopic signals, we did not observe a second set of signals in the NMR spectrum of aza-aspartame **9**, which may be due to rapid isomerization, or the presence of a single isomer. The authors tasted crystals of aza-aspartame **9** and came to the consensus that it was slightly bitter or tasteless.

## 3. Experimental

### General

Potassium carbonate, *tert*-butyl carbazate, DMF, *tert*-butyl bromoacetate, DIEA and 20% phosgene solution in toluene, all were purchased from Sigma-Aldrich (Oakville, ON, Canada). Anhydrous dichloromethane (DCM) was obtained by passage through a solvent filtration system (GlassContour, Irvine, CA, USA). EtOAc and hexane were purchased from Anachemia (Saint-Pierre, QC, Canada). Hexane was fractionally distilled prior to use for flash chromatography. Flash chromatography [9] was performed on 230–400 mesh silica gel; crude product was loaded as a concentrated solution in DCM onto a sand layer over the silica gel column, which was packed using the specified solvent system. Thin-layer chromatography was performed on silica gel 60 F254 plates from Merck™ (Oakville, ON, Canada). Melting points were made on a Gallankamp apparatus, and are uncorrected. Specific rotations, [α]_D_ values, were calculated from optical rotations measured at 20 °C in CHCl_3_ at the specified concentrations (*c* in mg/1 mL) using a 1-dm cell (*l*) on a PerkinElmer Polarimeter 341, using the general formula: [α]^20^_D_ = (100•*α*)/(*l*•*c*). Accurate mass measurements were performed on a LC-MSD instrument from Agilent technologies in positive electrospray ionization (ESI) mode at the Université de Montréal Mass Spectrometry facility. Hydrogen adducts [MH]^+^ were used for empirical formula confirmation. ^1^H-NMR spectra were measured in CDCl_3_ (7.26 ppm) or CD_3_OD (3.34 ppm). ^13^C-NMR spectra were measured in CDCl_3_ (77.36 ppm) or DMSO-*d_6_* (39.51 ppm). Coupling constant *J* values are measured in Hertz (Hz) and chemical shift values are reported in parts per million (ppm). Measurements were made on a Bruker NMR instrument. Infrared spectra were recorded in the neat on an ATR Bruker apparatus.

*tert-Butyl 2-(2-(tert-butoxy)-2-oxoethyl)hydrazine carboxylate* (**11**). A well stirred 0 °C solution of *tert*-butyl carbazate (2.6 g, 17.8 mmol) and K_2_CO_3_ (2.7 g, 19.6 mmol) in dry DMF (71 mL) was treated with *tert*-butyl bromoacetate (2.6 mL, 17.8 mmol) over 5 min, and allowed to warm to room temperature. The reaction mixture turned cloudy after a few hours. After stirring at room temperature for 55 h, the mixture was diluted with water (250 mL) and extracted with EtOAc (3 × 75 mL). The combined organic extracts were washed with water (100 mL) and brine (100 mL), and dried over MgSO_4_. The volatiles were evaporated to a residue, which was purified by flash chromatography [8] eluting with 20% ethyl acetate in hexanes. Evaporation of the collected fractions gave 1.02 g (23%) of carbazate **11** as a low melting colorless solid: R_f_ 0.28 (20% ethyl acetate in hexanes); IR (thin film, cm^−1^) ν 2978, 1702, 1534, 1477, 1392, 1364, 1244, 1143; ^1^H-NMR (300 MHz, CDCl_3_) δ 6.32 (s, 1H), 4.06 (s, 1H), 3.47 (s, 2H), 1.45 (d, *J* = 2.1 Hz, 9 H), 1.40 (d, *J* = 1.8 Hz, 9H); ^13^C-NMR (75 MHz, CDCl_3_) δ 170.5, 155.9, 81.8, 81.6, 80.5, 53.6, 27.2; HRMS calcd for C_11_H_23_N_2_O_4_ [MH]^+^ 247.1652, found 247.1650.

*β-tert-Butyl N-(Boc)Aza-aspartyl-phenylalanine methyl ester* (**13**). A solution of 150 mg (0.6 mmol) of *tert*-butyl 2-(2-(*tert*-butoxy)-2-oxoethyl)hydrazine carboxylate (**11**) in DCM (9 mL) under argon at 0 °C was treated drop-wise with a solution of 20% phosgene in toluene (5.25 mL, 1.22 mmol, CAUTION: phosgene is highly toxic and must be handled with great care in well-ventilated hoods), stirred for 30 min, and evaporated on a rotary evaporator equipped with a trap containing 1N NaOH. The residue was dissolved in DCM (3 mL), cooled to 0 °C, and treated dropwise with a solution of L-phenylalanine methyl ester hydrochloride (0.13 g, 0.61 mmol) and DIEA (1.74 mL, 1.22 mmol) in DCM (3 mL). The mixture was allowed to warm to room temperature with stirring for 9–10 h, and concentrated on a rotary evaporator to a residue, which was purified by chromatography on silica gel eluting with 30% EtOAc in hexane. Evaporation of the collected fractions gave protected dipeptide **13** (160 mg, 58%) as a colorless oil: R_f_ 0.59 (30% EtOAc-Hexanes); [α]^25^_D_ 1.51 (CHCl_3_, *c* 1.0); IR (thin film, cm^−1^) ν 2979, 1731, 1676, 1515, 1367, 1233, 1148; ^1^H-NMR (300 MHz, CDCl_3_) δ 7.19 (m, 3H), 7.05 (d, *J* = 5.4 Hz, 2H), 6.7 (s, 1H), 5.92 (d, *J* = 7.8 Hz, 1H), 5.22 (s, 1H), 4.69 (dd, *J* = 5.54 Hz, *J* = 7.8 Hz, 1H), 3.62 (s, 3H), 3.05 (d, *J* = 5.7 Hz, 2H), 1.45 (d, *J* = 2.1, 1H), 1.4 (d, *J* = 8.1, 18 H); ^13^C-NMR (75 MHz, CDCl_3_) δ 171.9, 169.2, 156.5, 154.2, 135.9, 129.5, 128.5, 126.8, 82.3, 54.5, 53.2, 51.8, 48.7, 38.2, 28.2; HRMS calcd. for C_22_H_33_N_3_NaO_7_ [M+Na]^+^ 474.2211, found 474.2214.

*Aza-aspartylphenylalanine methyl ester* (**9**). Protected dipeptide **13** (400 mg, 0.88 mmol) in DCM (20 mL) was exposed to HCl gas bubbles for 1 h, during which time a precipitate formed. The reaction mixture was concentrated on a rotary evaporator to a residue, which was purified by HPLC on a synergi RP-Polar column [100 × 21.20 mm, 4 micron] using a flow rate of 10 mL/min, eluting with a gradient of 50%–80% MeOH (1% formic acid, FA) in H_2_O (1% FA) over 12 min followed by 80% MeOH (1% FA) in H_2_O (1% FA) for 18 min. Evaporation of the collected fractions gave aza-aspartame **9** (70 mg, 21%) as colorless crystals: mp 168 °C; [α]^25^_D_ 14.1 (MeOH, *c* 1.0); IR (thin film cm^−1^) ν 3300, 2497, 1736, 1609, 1542, 1452, 1388, 1325, 1217, 1097; ^1^H-NMR (300 MHz, CD_3_OD) δ 7.15 (m, 5H), 4.46 (t, *J* = 6 Hz, 1H), 4.09 (dd, *J* = 3.6 Hz, *J* = 24.8 Hz, 2H), 3.58 (s, 3H), 2.99 (d, *J* = 6.3 Hz, 2H), 1.20 (s, 2H); ^13^C-NMR (175 MHz, DMSO-d_6_) δ 173.0, 171.7, 158.6, 137.4, 129.6, 128.8, 127.1, 54.7, 52.2, 51.0, 37.8; HRMS calcd. for C_13_H_18_N_3_O_5_ [MH]^+^ 296.1241, found 296.1238.

## 4. Conclusions

Aza-aspartame **9** was synthesized, crystallized, examined by X-ray analysis and tasted. In the solid state, the nitrogen of the semicarbazide was nearly planar, yet adopted the *R*-configuration. On tasting, the authors concluded that aza-aspartame **9** was slightly bitter or tasteless. Notably, the threshold for bitter compounds generally appears to be lower than that of sweet compounds [7]. The absence of a sweet taste may be due to the flat sp^2^ hybridization of the nitrogen in the urea moiety of **9** as well as inability for the sp^3^ nitrogen to exhibit dynamic chirality preventing recognition by the receptors for sweetness. 

## References

[1] Cox C., Lectka T. (1998). Solvent effect on the barrier to rotation in carbamates. J. Org. Chem..

[2] Noguchi T., Shimazawa R., Nagasawa K., Hashimoto Y. (2002). Thalidomide and its analogues as cyclooxygenase inhibitors. Bioorg. Med. Chem. Lett..

[3] Sauerberg P., Chen J., WoldeMussie E., Rapoport H. (1989). Cyclic carbamate analogues of pilocarpine. J. Med. Chem..

[4] Drake M.V., O’Donnell J.J., Polansky J.R. (1986). Isopilocarpine binding to muscarinic cholinergic receptors. J. Pharm. Sci..

[5] Proulx C., Sabatino D., Hopewell R., Spiegel J., García-Ramos Y., Lubell W.D. (2011). Azapeptides and their therapeutic potential. Future Med. Chem..

[6] Boeglin D., Xiang Z., Sorenson N.B., Wood M.S., Haskell-Luevano C., Lubell W.D. (2006). Aza-scanning of the potent melanocortin receptor agonist Ac-His-D-Phe-Arg-Trp-NH_2_. Chem. Biol. Drug Des..

[7] Temussi P.A. (2012). The good taste of peptides. J. Pept. Sci..

[8] Still W.C., Kahn M., Mitra A. (1978). Rapid chromatographic technique for preparative separations with moderate resolution. J. Org. Chem..

[9] Guguta C., Meekes H., de Gelder R. (2006). Crystal structure of aspartame anhydrate from powder diffraction data. Cryst. Growth Des..

[10] The Cabridg (CCDC 972051). www.ccdc.cam.ac.uk.

